# Regional citrate anticoagulation versus low molecular weight heparin anticoagulation for continuous venovenous hemofiltration in patients with severe hypercalcemia: a retrospective cohort study

**DOI:** 10.1080/0886022X.2020.1795879

**Published:** 2020-07-28

**Authors:** Yan Yu, Ming Bai, Zhang Wei, Lijuan Zhao, Yangping Li, Feng Ma, Shiren Sun

**Affiliations:** Department of Nephrology, Xijing Hospital, Fourth Military Medical University, Shaanxi, China

**Keywords:** Severe hypercalcemia, continuous venovenous hemofiltration, regional citrate anticoagulation, low molecular weight heparin anticoagulation, acute kidney injury, serum calcium reduction rate

## Abstract

**Purpose:**

We conducted a retrospective study to evaluate the efficacy and safety of regional citrate anticoagulation (RCA) versus those of low molecular weight heparin (LMWH) anticoagulation for CVVH in severe hypercalcemia patients.

**Methods:**

Between January 2014 and May 2019, 33 severe hypercalcemia patients underwent CVVH. Patients were divided into the RCA and LMWH groups. Calcium-free replacement solution was used. Serum total calcium reduction rate (RRSeCa), filter lifespan, bleeding, totCa/ionCa ratio, citrate accumulation, and catheter occlusion were evaluated as outcomes.

**Results:**

RCA and LMWH were employed for CVVH in 14 and 43 filters, respectively. RRSeCa was not significantly different between the LMWH and RCA groups (*p* = .320), but RCA-CVVH was more effective in reducing ionized calcium at half of the time points (*p* < .05). RCA significantly prolonged the median filter lifespan (>72 h vs. 24.0 h [IQR, 15.0–26.0], *p* = .012). The incidence of filter failure was 55.8% (24/43) in the LMWH group and 21.4% (3/14) in the RCA group (*p* = .033). The adjusted results demonstrated that RCA could significantly reduce the risk of filter failure (*p* = .043, 95% CI 0.059–0.957, HR = 0.238). No citrate accumulation or bleeding episodes were observed in the RCA-CVVH group. Seven bleeding episodes (7/43, 16.3%) occurred in the LMWH-CVVH group.

**Conclusions:**

In patients with severe hypercalcemia who underwent CVVH, RCA more effectively decreased calcium levels and had a superior filter lifespan and no obvious adverse events compared with LMWH. Further prospective, randomized, controlled studies are warranted to obtain robust evidence.

## Introduction

Hypercalcemia is an electrolyte disorder commonly seen in routine clinical practice and accounts for approximately 0.6% of all emergency hospital admissions, with a prevalence rate of 1–7/1000 in the general population [1,2]. Primary hyperparathyroidism and malignancy are the most common causes for hypercalcemia [2,3]. Although severe hypercalcemia (‘hypercalcemic crisis’) only occurs 1.6–6.7% in hypercalcemia patients, it leads to a 14-fold increase in acute kidney injury (AKI) risk [4,5] and is associated with a mortality that ranges from 15% to 100% [6–10]. Nausea, vomiting, weakness, arrhythmia, and disorientation are the major symptoms of hypercalcemia [1,2]. Conventional treatments for hypercalcemia include intravenous fluids, loop diuretics, steroids, calcitonin, and bisphosphonates [11]. To treat the primary disease, efforts should include surgery, chemotherapy, and/or radiation [12]. For patients with severe hypercalcemia, a poor response to conservative treatment, renal dysfunction, and heart failure, intermittent hemodialysis treatment is recommended [1,5,11,13]. However, intermittent hemodialysis with calcium-free/low-calcium dialysate might result in rebound hypercalcemia, hypovolemia, and hypotension [4,14]. Continuous renal replacement therapy (CRRT), especially continuous venovenous hemofiltration (CVVH), is the most commonly used hemodialysis modality for critically ill patients, especially for those with hemodynamic instability [15,16]. Several case reports have shown that CRRT could successfully reduce serum calcium concentration with stable hemodynamics [17–20].

During CVVH treatment, premature clotting in the extracorporeal circuit shortens the lifespan of the filter and catheter, reduces the effectiveness of CVVH, causes blood loss, and increases the medical cost and the staff’s workload [21,22]. The major intervention to maintain patency of the extracorporeal circuit is anticoagulation. Numerous clinical studies have demonstrated that regional citrate anticoagulation (RCA) for CRRT could prolong the filter lifespan and decrease the bleeding risk compared to heparin or low molecular weight heparin (LMWH) anticoagulation [23–26]. The Kidney Disease Improving Global Outcomes (KDIGO) guidelines recommended RCA as the first choice for CRRT in patients without citrate contraindications [15].

As we know, calcium is a cofactor in the coagulation cascade, and citrate exerts its anticoagulant effect by chelating ionized calcium (ionCa; ideally target for an ionCa level was < 0.4 mmol/L) in the extracorporeal circuit and therefore inhibits the clotting cascade [25]. Theoretically, severe hypercalcemia could reduce the efficacy of citrate anticoagulation and potentially lead to a shortened filter lifespan. In addition, RCA directly impacts calcium equilibrium in patients, even leading to a high risk of bone resorption and secondary hyperparathyroidism [27,28]. The standard operating procedure for blood purification in China suggested that patients with severe liver failure, severe hypoxia, reduced organ perfusion and hypercalcemia should not accept RCA for CRRT [29]. Most likely, clinicians in other countries were confused on RCA for CRRT in hypernatremia patients as well [30]. Therefore, during our clinical practice, the choice of an appropriate anticoagulant for CVVH in severe hypercalcemia patients is challenging, especially for patients with contraindications to systemic anticoagulation, including patients with an increased bleeding risk. For these patients, RCA is definitely a safer anticoagulation strategy than LMWH. However, to the best of our knowledge, no cohort study has assessed the safety and efficacy of RCA for CVVH in severe hypercalcemia patients, and no study has compared LMWH anticoagulation with RCA anticoagulation in hypercalcemia patients undergoing CVVH. Therefore, the purpose of our present study was to assess the efficacy and safety of RCA-CVVH versus LMWH anticoagulation in severe hypercalcemia patients.

## Methods

### Study design and patient selection

Our present study was a retrospective cohort study from a single center that treats approximately 2000 critically ill patients who undergo CVVH per year. Patients with severe hypercalcemia who received CVVH therapy in our center between January 2014 and May 2019 were considered candidates. In our clinical practice, the occurrence of severe hypercalcemia resisted to conservative treatment, other severe electrolyte disorder, progressive acute kidney injury, fluid overload, and severe acid-base disorder were considered the indication for CRRT in patients with severe hypercalcemia. Severe hypercalcemia was defined as a total serum calcium level higher than 3.5 mmol/L or higher than 3.0 mmol/L with obvious symptoms related to hypercalcemia, such as nausea, weakness, disorientation, and arrhythmia [1,2,4]. The conservative treatment for hypercalcemia included fluid resuscitation, loop diuretics, treatment of the cause of hypercalcemia, steroids, calcitonin, and bisphosphonates. Patients meeting any of the following criteria were excluded: need for therapeutic anticoagulation, surgery within 24 h before CVVH, CVVH performed by using arteriovenous fistula, pregnancy, CVVH with no anticoagulation, and interruption to CVVH due to an examination or operation. According to the anticoagulation strategy for CVVH, the included patients were divided into the RCA group and LMWH-anticoagulation group.

Our present study was performed in accordance with the Declaration of Helsinki and approved by the ethics committee of our hospital (KY20192101). The requirement for patient consent was waived due to the retrospective nature of the study. Before CVVH treatment, all of the patients received detailed information about the advantages and disadvantages of CVVH treatment and provided written informed consent.

### Characteristics of the CVVH protocol

Temporary vascular access was created by inserting a 13.5 Fr dual-lumen catheter into the femoral vein or jugular vein. The use of CVVH was decided by the doctor in charge according to his/her clinical experiences. CVVH was performed by using the Prismaflex device with an M100 Set system (Gambro, Sweden), which has an effective membrane area of 0.9 m^2^, or AV600S (Frensius, German), which has a membrane area of 1.2 m^2^. The replacement fluid was infused at 50% predilution and 50% post-dilution at a speed of 2 L/h.

Calcium-free replacement solution was used. In the RCA group, the initial blood flow rate was 150–180 mL/min, and the dose of 4% trisodium citrate was 200–300 mL/h (2.52–4.53 mmol per liter blood) to decrease calcium levels, with or without 10% calcium gluconate for calcium supplementation. The blood flow or RCA dose was modified to achieve a postfilter ionCa level between 0.25 and 0.56 mmol/L. Citrate dose was increased by 10 mL/h or blood flow was decreased by 10 mL/min on the condition of the postfilter ionCa >0.56 mmol/L, and citrate dose was decreased by 10 mL/h or blood flow was increased by 10 mL/min on the condition of postfilter ionCa < 0.25 mmol/L. Calcium supplement was not given on the condition of systemic ionCa concentration > 1.3 mmol/L. During the CVVH treatment, calcium supplement (10% calcium gluconate) was increased by 10 mL/h on the condition of systemic ionCa < 1.0 mmol/L, and calcium supplement was decreased by 10 mL/h on the condition of systemic ionCa > 1.30 mmol/L. In the LMWH group, the initial blood flow rate was 200 mL/min without supplementation with a 10% calcium gluconate solution. The patients received an intravenous bolus of nadroparin 1500–3500 IU at the initiation of CVVH, followed by 500–1000 IU per 4 h. And, the LMWH dose was adjusted depending on the patient’s body weight and coagulation parameters.

Intensive metabolic monitoring, including postfilter ionCa in the RCA group, ionized and total serum calcium, sodium and potassium levels, and blood gas analyses (pH, pCO_2_, HCO_3_^−^, and BE) were performed every 4 or 8 h in both groups. CVVH treatment was continued until hypercalcemia was corrected, treatment was abandoned, or the patient died. The filter was routinely replaced every 72 h even without filter failure. Filter pressures, circuit clotting, and bleeding episodes were monitored continuously.

### Data collection

Data were retrieved from the electronic medical records of our hospital. Baseline characteristics, including demographic, clinical, and biochemical data, APACHE II score, and SOFA score at the beginning of CVVH were recorded. The other parameters assessed during CVVH treatment are as follows: mechanical ventilation, vasopressor dependency, laboratory parameters, anticoagulation method, vascular access, ultrafiltration rate, blood flow rate, duration of CVVH (hours), runtime of the filter (hours), circuit coagulation, filter numbers, reason for filter exchange, and filtration fraction. The blood gas analyses, blood cell analysis, liver function, renal function, electrolyte test, and coagulation function tests results and treatment-related complications (filter failure, catheter occlusion, bleeding, citrate accumulation, acid-base disorders) during CVVH treatment were recorded.

### Outcomes and definitions

The serum calcium reduction rate (RRSeCa) was calculated as follows: RRSeCa (mmol/L/h) = change of serum calcium concentration (mmol/L)/treatment time (hours). The filter lifespan was defined as the time from the beginning to the termination of a CVVH cycle. The reasons of CVVH cycle termination included the achievement of treatment target, filter failure, patient death and the upper time limitation of a filter (72 h). Filter failure was defined as TMP (transmembrane pressure) ≥ 300 mmHg or circuit clotting. The filtration fraction (FF) was calculated as follows: FF (%) = ultrafiltration rate/blood flow rate (ml/min)*(1-HCT)*60.

Safety was assessed by the frequency of adverse events, which were defined as bleeding, catheter occlusion, totCa/ionCa > 2.5, acidosis (pH < 7.35), and alkalosis (pH > 7.45). Metabolic acidosis with an elevated anion gap, reduced ionCa, elevated total calcium, and a calcium ratio (totCa/ionCa) > 2.5 was considered citrate accumulation [31,32].

### Statistical analysis

Continuous variables are presented as the means ± standard deviations (SD) or medians with interquartile ranges (IQRs), and categorical variables are presented as event numbers and percentages (%). We performed a normality test first, and a *t*-test was used for data with a normal distribution. The Mann–Whitney rank test was used for data with a non-normal distribution. The χ^2^ test or Fisher’s exact test were employed for categorical variables. The estimated median filter lifespan was assessed by the Kaplan–Meier survival curve and compared using log-rank test, and the risk factors for time-dependent outcome were identified by the Cox regression model. The covariates included in the univariate analysis were age, sex, comorbidities, temporary vascular access site, filter type, filtration fraction (FF), baseline platelet counts, hemoglobin, prothrombin time (PT), activated partial thromboplastin time (APTT), serum Ca, serum creatinine, blood urea nitrogen, serum albumin, serum total bilirubin and anticoagulation strategy. The baseline data were measured before the start of each CVVH session. Variables with *p* < .05 in the univariate analysis and clinically important variables that were not identified as a risk factor in the univariate analysis, including FF, platelet counts and APTT, were included in the multivariate Cox regression analyses. The filter failure percentages were calculated by the number of filters with filter failure to the total number of the included filters. All tests were 2 sided, and a *p* value < .05 was considered statistically significant. Statistical analysis was performed by using IBM SPSS (Version 21.0 for Windows, Armonk, New York, 2012).

## Results

### Baseline characteristics

**Figure 1.**
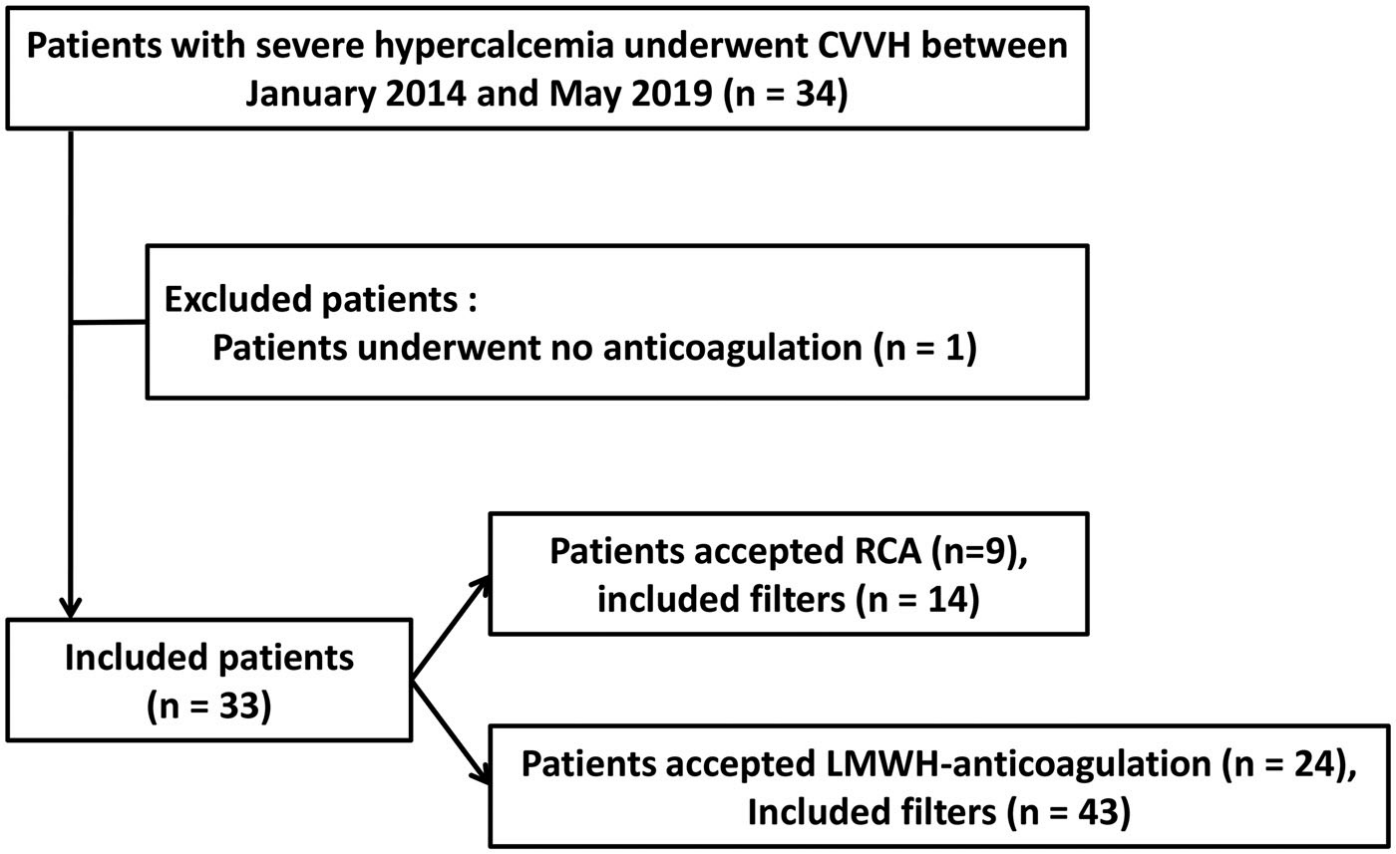
Patient inclusion flowchart.

Between January 2014 and May 2019, 34 consecutive patients with severe hypercalcemia who received CVVH therapy were enrolled in our center. Of these patients, one patient was excluded based on the exclusion criteria. Finally, 33 patients were included. Of the included patients, 9 patients underwent RCA-CVVH with 14 filters, and 24 patients underwent LMWH-anticoagulation CVVH with 43 filters (Figure 1). Fifteen patients (15/33, 45.5%) were treated with CVVH for repeat hypercalcemia during hospitalization.

The baseline characteristics of the included patients are described in Table 1. Overall, 75.8% of the patients were male with a mean age of 56.3 ± 17.2 years. Patients in the LMWH-anticoagulation group were older than those in the RCA group (60.0 ± 14.8 vs. 46.4 ± 20.2, *p* = .042). The median serum calcium was 3.78 ± 0.52 mmol/L before CVVH. The two groups did not have significantly different serum calcium levels or remaining baseline characteristics (Table 1). The causes of hypercalcemia were malignancy (including multiple myeloma, non-Hodgkin lymphoma, hepatoma, esophageal cancer and acute leukemia) in 78.7% of the patients and primary parathyroid adenoma in 21.2% of the patients. AKI (27/33, 81.8%), somnolence (6/33, 18.2%), and arrhythmia (3/33, 9.1%) were the most common serious symptoms of hypercalcemia, followed by coma (2/33, 6.1%).

**Table 1.** Baseline characteristics of the included patients.

Variables	Total (*n* = 33)	RCA (*n* = 9)	LMWH-anticoagulation (*n* = 24)	*p*-Value
Male, n (%)	25 (75.8)	6 (66.7)	19 (79.2)	.651
Age, years	56.3 ± 17.2	46.4 ± 20.2	60.0 ± 14.8	.042
APACHE II score	13.9 ± 4.9	15.2 ± 5.8	13.3 ± 4.5	.330
SOFA score	3.73 ± 2.14	4.33 ± 2.78	3.50 ± 1.87	.327
GCS score	14.3 ± 1.0	14.11 ± 1.17	14.42 ± 0.97	.452
MAP, mmHg	91.4 ± 11.5	90.5 ± 10.3	91.8 ± 12.1	.789
Vasopressor dependency, n (%)	1 (3.0)	0 (4.2)	1 (0)	1.000
Mechanical ventilation, n (%)	1 (3.0)	0 (4.2)	1 (0)	1.000
Etiology of hypercalcemia				
Multiple myeloma, yes, n (%)	18 (54.5)	4 (44.4)	14 (58.3)	.697
Other malignancy, yes, n (%)	8 (24.2)	2 (22.2)	6 (25.0)	1.000
Primary hyperparathyroidism, yes, n (%)	7 (21.2)	3 (33.3)	4 (16.7)	.358
Serum calcium, mmol/L	3.78 ± 0.52	3.67 ± 0.48	3.83 ± 0.54	.466
Serum potassium, mmol/L	3.82 ± 0.64	4.02 ± 0.73	3.75 ± 0.60	.286
Serum natrium, mmol/L	140.5 ± 0.64	138.1 ± 4.32	141.4 ± 8.67	.275
BUN, mmol/L,	17.5 ± 10.1	20.2 ± 10.6	16.5 ± 9.9	.357
Serum Creatinine, μmol/L	319.8 ± 240.2	341.7 ± 308.4	311.6 ± 216.6	.754
CysC, mg/dL	2.89 ± 1.54	2.70 ± 1.75	2.96 ± 1.48	.677
Serum uric acid, μmolL	475 ± 227.7	401.2 ± 281.6	499.6 ± 208.9	.332
ALT, IU/L	26.4 ± 24.7	33.2 ± 30.8	23.9 ± 22.2	.341
AST, IU/L	39.4 ± 45.8	45.1 ± 50.8	37.3 ± 44.8	.671
Serum ALB, g/L	34.1 ± 6.6	33.78 ± 6.3	34.22 ± 6.85	.872
Serum total bilirubin, μmol/L	14.2 ± 9.8	17.1 ± 13.5	13.1 ± 8.1	.310
Platelet, 109/L	160.9 ± 78.5	180.2 ± 122.8	153.7 ± 55.9	.395
Hemoglobin, g/L	102.5 ± 33.3	101.3 ± 37.0	102.9 ± 32.6	.903
PT, s	12.4 ± 1.6	12.7 ± 2.0	12.2 ± 1.4	.455
APTT, s	32.3 ± 27.6	26.3 ± 7.4	34.6 ± 32.0	.453
PTA, %	81.6 ± 21.3	85.4 ± 22.4	80.2 ± 21.2	.545
INR	1.07 ± 0.14	1.07 ± 0.15	1.07 ± 0.13	.963
NT-Pro BNP	3639.4 ± 8050.2	2091.5 ± 1369.9	4131.9 ± 9209.7	.569
AKI yes, n (%)	27 (81.8)	7 (77.8)	20 (83.3)	1.000
Hyperkalemia, yes, n (%)	1 (3.0)	1 (11.1)	0 (0)	.273
CVVH indications				
Progressive AKI, n (%)	27 (81.8)	7 (77.8)	20 (83.3)	1.000
Volume overload, n (%)	6 (18.2)	1 (11.1)	5 (20.8)	1.000
Hypernatremia, n (%)	5 (15.2)	1 (11.1)	4 (16.7)	1.000
Acidosis, yes, n (%)	3 (9.1)	1 (11.1)	2 (8.3)	1.000
Alkalosis, yes, n (%)	3 (9.1)	0 (0)	3 (12.5)	.545
Hospital stay, days	12.9 ± 9.5	14.7 ± 10.3	12.3 ± 9.2	.529
ICU stay, days	2.0 ± 3.1	2.6 ± 3.2	1.8 ± 3.1	.536

RCA: Regional citrate anticoagulation; CVVH: continuous venovenous hemofiltration; APTT: activated partial thromboplastin time; PTA: prothrombin time activity; INR: international normalized ratio; BUN: blood urea nitrogen; AKI: Acute kidney injury; NT-Pro BNP: N-terminal pro-brain natriuretic peptide. Unless indicated otherwise: data are presented as the mean ± standard deviation (SD). Other malignancy includes non-hodgkin lymphoma: hepatoma: esophagus cancer: acute leukemia.

### Efficacy outcomes

#### Correction of hypercalcemia

The mean serum calcium concentrations before CVVH treatment were 3.67 ± 0.48 mmol/L and 3.83 ± 0.54 mmol/L in the RCA and LMWH-anticoagulation groups, respectively (*p* = .466). The initial median calcium infusion rate used for RCA compensation was 1.33 mmol/hour (IQR, 0–2.23). After 4 h of CVVH, the mean serum calcium concentration was effectively reduced to 2.97 ± 0.39 mmol/L and 2.85 ± 0.52 mmol/L in the RCA and LMWH-anticoagulation groups, respectively (*p* = .524). At the end of CVVH, the mean serum calcium concentrations were 2.46 ± 0.56 mmol/L and 2.44 ± 0.43 mmol/L, respectively (*p* = .923). The mean RRSeCa was 0.037 ± 0.019 mmol/L/h in the RCA group and 0.054 ± 0.049 mmol/L/h in the LMWH-anticoagulation group (*p* = .320, Table 2). The average total calcium levels at 24 h after CVVH were 2.69 ± 0.63 mmol/L and 2.67 ± 0.32 mmol/L in the RCA and LMWH-anticoagulation groups, respectively (*p* = .892).

**Table 2.** Parameters in CVVH sessions.

Parameters	RCA (*n* = 14)	LMWH-anticoagulation (*n* = 43)	*p*-Value
M100/AV600, n (%)	12 (85.7) / 2 (14.3)	38 (88.4) /5 (11.6)	1.000
Initiation blood flow, ml/ min (IQR)	180 (180–180)	200 (200–200)	–
Dose of 4% trisodium citrate, ml/ h (IQR)			
Initiation	200 (200–200)	–	–
CVVH for 4 h	200 (200–200)	–	–
CVVH for 8 h	200 (200–200)	–	–
CVVH for 12 h	200 (200–200)	–	–
CVVH for 24 h	200 (200–200)	–	–
End of CVVH	200 (200–200)	–	–
Initiation LMWH dose, IU/kg, mean ± SD	–	38.8 ± 14.1	–
Filtration fraction, %, mean ± SD	13.23 ± 4.28	11.30 ± 2.93	.135
Mean hypercalcemia correction rates, mmol/L/h, mean ± SD	0.037 ± 0.019	0.054 ± 0.049	.320
Mean ionized calcium reduction rate, mmol/L/h, mean ± SD	0.057 ± 0.012	0.048 ± 0.019	.219
Supplement of 10% calcium gluconate, mmol/hr (IQR)			
Start of CVVH	1.33 (0–2.23)	0	–
CVVH for 4 h	0.67 (0–2.23)	0	–
CVVH for 8 h	0 (0–1.78)	0	–
CVVH for 12 h	0 (0–1.34)	0 (0–0.50)	–
CVVH for 24 h	0 (0–1.34)	0	–
Reason for the termination of CVVH cycle			
TMP ≥ 300, mmHg, n (%)	3 (21.4)	16 (37.2)	.343
Filter clotting, n (%)	3 (21.4)	24 (55.8)	.033
Achievement of treatment goal, n (%)	9 (64.3)	26 (60.5)	1.000
Filter replacement at 72 h, n (%)	1 (7.1)	0	.246

CVVH: continuous venovenous hemofiltration; RCA: Regional citrate anticoagulation; LMWH: Low molecular weight heparin.

**Figure 2.**
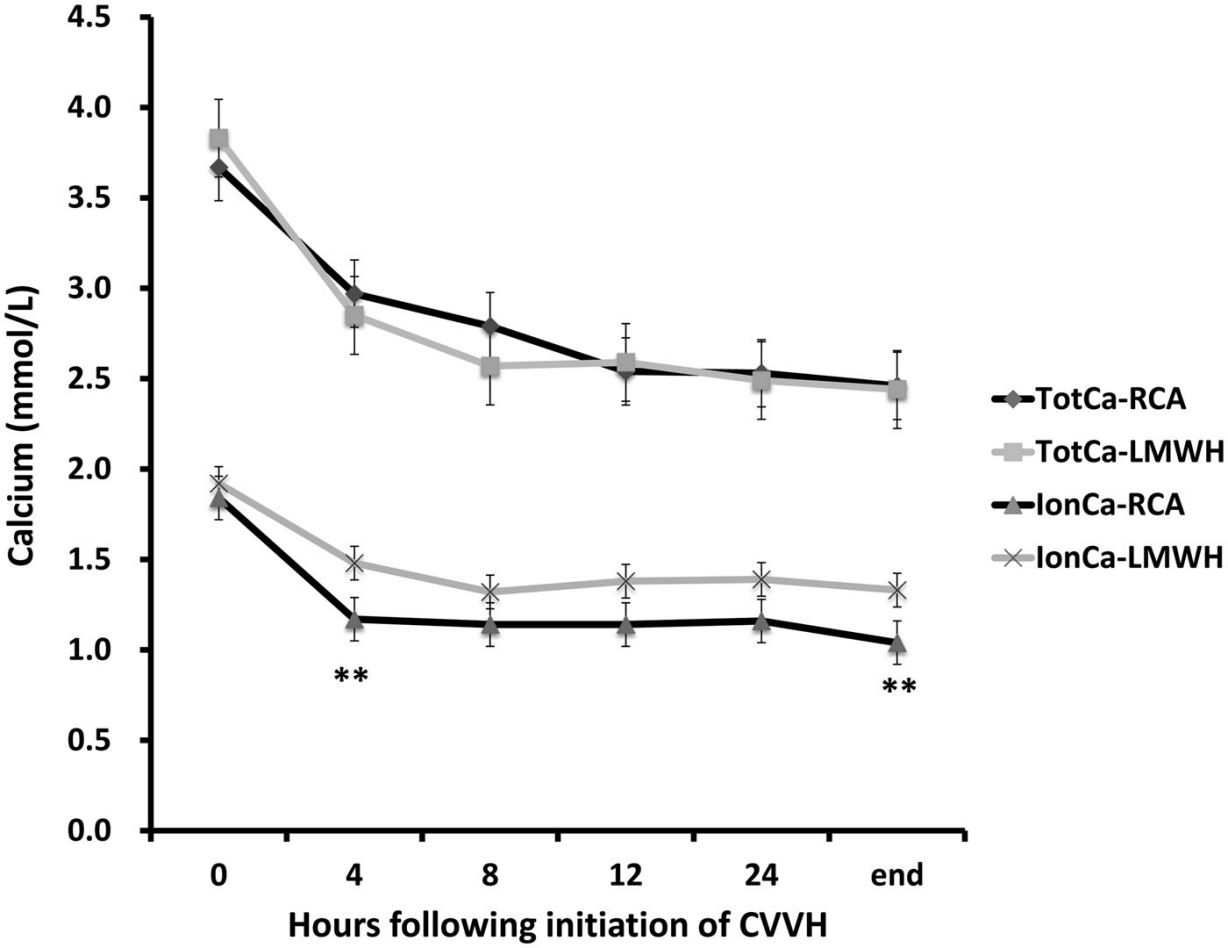
Course of serum calcium concentrations during CVVH therapy. **The ionCa levels after 4 h (*P* = 0.015) and the end of CVVH (*p* = .029) were significantly lower in the RCA group than in the LMWH-anticoagulation group.

Figure 2 further shows the respective total calcium and systemic ionCa levels during CVVH. The mean ionCa concentrations before CVVH treatment were 1.79 ± 0.23 mmol/L and 1.84 ± 0.26 mmol/L in the RCA and LMWH-anticoagulation groups, respectively (*p* = .486). During CVVH treatment, the ionCa levels tended to be lower in the RCA group, and the ionCa levels after 4 h (1.19 ± 0.27 mmol/L vs. 1.48 ± 0.39 mmol/L, *p* = .015) and the end of CVVH (1.04 ± 0.25 mmol/L vs. 1.33 ± 0.28 mmol/L, *p* = .029) were significantly lower in the RCA group than in the LMWH-anticoagulation group (Figure 2). The mean ionCa reduction rates in the first 4 h were 0.138 ± 0.098 mmol/L/h in the RCA group and 0.079 ± 0.033 mmol/L/h in the LMWH-anticoagulation group (*p* = .033) and those after all CVVH sessions were 0.57 ± 0.012 mmol/L/h and 0.048 ± 0.019 mmol/L/h, respectively.

#### Filter lifespan

**Figure 3.**
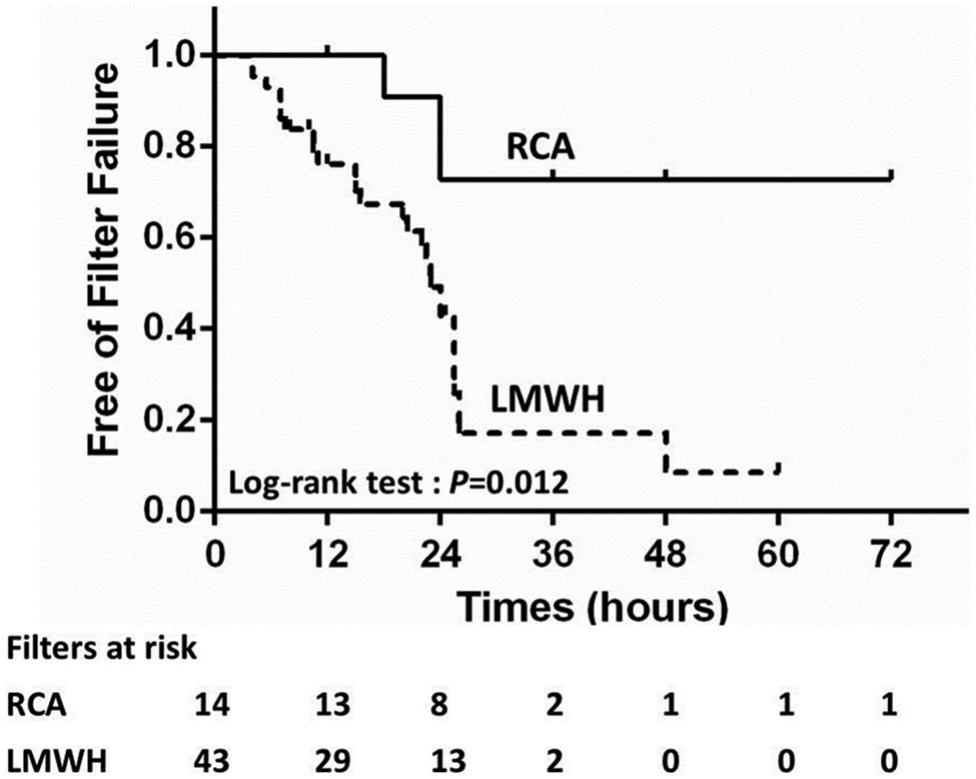
Survival curves of the filters between the RCA and LMWH-anticoagulation groups in the cohort.

The raw median lifespan time were 24 h (IQR, 16.5–27) and 20 h (IQR, 10.5–24) in the RCA group and LMWH-anticoagulation group, respectively (*p* = .033). In the LMWH-anticoagulation group, patients received an intravenous bolus of nadroparin at 38.8 ± 14.1 IU/kg at the initiation of CVVH, followed by 12.5 ± 4.8 IU/kg/4 h, and the estimated median filter lifespan was 24.0 h (IQR, 15.0–26.0). The 4% citrate rate was 200 mL/h during the whole CVVH treatment in most of the patients (87%) in the RCA group. Only two patients had adjusted citrate dose after the first test of the postfilter ionCa during the CVVH treatment. One patient had 210 mL/h and the other patient had 250 mL/h citrate for four hours, and were reduced to 200 mL/h during the remaining period of the CVVH cycle. The average postfilter ionCa level at 2-h of CVVH was 0.52 ± 0.22 mmol/L. The estimated median filter lifespan of the RCA group was longer than 72 h. The shortest filter lifespan observed was 18 h, with a total calcium level of 4.0 mmol/L. For anticoagulation, a concentration of 2.52 (one patient 4.53) mmol citrate per liter blood was given at the initiation of CVVH, and the citrate dose increased by 0.3 mmol/L blood or blood flow decreased by 30 mL/h when the postfilter ionCa level was >0.56 mmol/L. No episodes of postfilter ionCa level <0.25 mmol/L were observed. The average rate of decline in ionCa was 51.3% after 2 h of CVVH. The RCA group had a significantly longer median filter lifespan than the LMWH-anticoagulation group (*p* = .012, Figure 3). The incidence of filter failure was 55.8% (24/43) in the LMWH-anticoagulation group and 21.4% (3/14) in the RCA group (*p* = .033, Table 3).

**Table 3.** Efficacy and safety outcomes during CVVH treatment.

	RCA (*n* = 14)	LMWH-anticoagulation (*n* = 43)	*p*-Value
Efficacy			
Estimated median filter lifespan, h (IQR)	>72	24.0 (15.0–26.0)	.012
Filter failure percent, n (%)	21.4 (3/14)	55.8% (24/43)	.033
Safety			
Bleeding, n (%)	0 (0)	7 (16.3)	.176
Blood transfusion, n (%)	2 (14.3)	7 (16.3)	1.000
TotCa/ionCa > 2.5, n (%)	1 (7.14)	3 (6.97)	1.000
Citrate accumulation, n (%)	0 (0)	–	–
Acidosis, pH < 7.35, n (%)	0 (0)	0 (0)	–
Alkalosis, pH > 7.45, n (%)	1 (7.14)	3 (6.97)	1.000

TotCa: total calcium; ionCa: ionized calcium.

In the univariate Cox analysis, the following factors had a statistically significant influence on filter lifespan: RCA (*p* = .023), vascular access site (*p* = .024), and baseline hemoglobin (*p* = .019). Multivariate Cox regression analyses revealed that the risk of filter failure was significantly reduced by RCA (*p* = .028, 95% CI 0.076–0.859, HR = 0.255). Moreover, after adjusting for clinically significant indicators (vascular access site, FF, PLT, and APTT), RCA significantly reduced the risk of filter failure (HR = 0.238, 95% CI 0.059–0.957, *p* = .043, Table 4).

**Table 4.** Predictors of filter failure in patients with severe hypercalcemia underwent CVVH.

		Univariate Cox regression			Multivariate Cox regression			Multivariate Cox regression adjusted the important clinical parameters		
		HR	95%CI	p Value	HR	95%CI	p Value	HR	95%CI	p Value
Anticoagulation strategy (RAC vs LMWH-anticoagulation)		0.247	0.074–0.826	.023	0.255	0.076–0.859	.028	0.238	0.059–0.957	.043
Filter Type (M100 vs AV600)		0.543	0.128–2.303	.408						
Vascular access site		2.529	1.132–5.649	.024	1.195	0.835–4.389	.125			
Baseline PLT		1.003	0.998–1.007	.267				1.001	0.994–1.008	.815
Baseline APTT		0.985	0.948–1.023	.429				0.984	0.958–1.011	.247
Baseline INR		1.073	0.796–1.445	.643						
Baseline HBG		1.015	1.002–1.027	.019	1.014	1.000–1.028	.052	1.015	0.999–1.032	.062
Baseline creatinine		0.998	0.995–1.000	.070						
Baseline Ca		1.396	0.673–2.895	.370						
Filtration fraction		0.977	0.868–1.099	.697				0.993	0.853–1.157	.933

RCA: Regional citrate anticoagulation; CVVH: continuous venovenous hemofiltration; PLT: platelet; APTT: activated partial thromboplastin time; INR: international normalized ratio; HBG: hemoglobin: Ca: calcium.

### Safety outcomes

#### Bleeding

Seven bleeding episodes (7/43, 16.3%) occurred in the LMWH-anticoagulation group, and no bleeding episodes were observed in the RCA group (*p* = .176). There was no difference in the blood transfusion requirement during CVVH treatment between the two groups (14.3% vs. 16.3%, *p* = 1.000).

#### totCa/ionCa and citrate accumulation

A transient totCa/ionCa > 2.5 (2.51 at 36-h and 2.58 at 48-h during CVVH) was observed in one patient in the RCA group. No metabolic acidosis, elevated anion gap or calcium elevation, and clinical symptoms of citrate accumulation (tremor, convulsions, and new arrhythmias) was observed. According to the citrate accumulation criteria, citrate accumulation was not diagnosed for this patient. Therefore, no change was made on the citrate dose and blood flow. All of the totCa/ionCa ratios recovered during the remaining CVVH period.

#### Other outcomes

No acidosis (pH < 7.35) was observed in either group. There was no significant difference in alkalosis episodes during CVVH treatment (7.14% vs. 6.97%, *p* = 1.000) between the two groups (Table 3). In addition, no catheter-related infections or catheter dysfunction were observed in either group. There were no significant changes in systolic blood pressure before and after CVVH treatment (119.3 ± 14.6 vs. 124.0 ± 18.6, *p* = .504; 115.2 ± 17.1 vs. 118.3 ± 21.4, *p* = .698) in either group, and there were no significant differences in diastolic blood pressure and mean arterial pressure between the two groups at the beginning and at the end of CVVH. The GCS score was significantly higher at the end of CVVH than at the beginning of CVVH in both groups (14.7 ± 0.6 vs. 14.3 ± 1.0, *p* = .003).

#### In-hospital mortality

The in-hospital mortality rates were 11.1% (1/9) and 16.7% (4/24) in the RCA and LMWH-anticoagulation groups, respectively (*p* = 1.000).

## Discussion

Studies on the treatment of severe hypercalcemia with CVVH are limited to anecdotes. To the best of our knowledge, our present study is the first cohort study to evaluate the efficacy and safety of RCA versus those of LMWH anticoagulation for CVVH in severe hypercalcemia patients. Our present study has several findings. First, RCA CVVH could reduce serum calcium more efficiently than LMWH-anticoagulation CVVH in hypercalcemia patients. Second, the use of RCA for CVVH could significantly increase the filter lifespan. Third, the use of RCA for CVVH did not significantly increase citrate accumulation, alkalosis, acidosis, or catheter occlusion and tended to decrease the risk of bleeding compared with the use of LMWH for CVVH.

There are controversial opinions on the use of RCA during CVVH therapy in severe hypercalcemia patients [15]. Kindgen-milles reported one case and Gradwohl *et al.* showed 4 cases in which RCA-CRRT could safely control hypercalcemia [18,19]. In our study, both RCA-CVVH and LMWH-CVVH effectively and stably reduced the serum calcium concentration. And, we observed that RCA-CVVH was more efficient in reducing ionCa than LMWH-CVVH. After 4 h of CVVH, the serum total calcium was effectively reduced in both groups, and at the end of CVVH, the serum total calcium was stably controlled to normal levels. Compared to the LMWH group, the RCA group had consistently lower ionCa levels, and the difference was significant after 4 h of CVVH and at the end of CVVH. During RCA CVVH, citrate directly chelated the ionCa, and approximately 50% of the citrate-calcium complexes were cleared by the hemofilter [33], which should be one of the reasons of the increased efficacy of ionCa in the RCA group. Additionally, the citrate entered the body chelate the ionCa in the systemic circulation, which should be another reasons of the increased reduction of ionCa in the RCA group.

Additionally, no rapid rebound hypercalcemia was observed after CVVH treatment in both groups. Theoretically, calcium re-distributes quickly among the tissue and blood. CVVH treatment continuously clear the serum calcium as well as the calcium released by the tissue. Therefore, CVVH could maintain the stability of the serum calcium concentration for a longer term. Calcium-free intermittent hemodialysis could reduced the serum calcium concentration as well [3,8]. However, after the 4 h calcium-free intermittent hemodialysis, the re-distribution of the calcium most likely would cause quickly rebound of the serum calcium level.

The results of our present study suggested that RCA was more effective than LMWH in prolonging the filter lifespan in severe hypercalcemia patients who underwent CVVH. These results were consistent with previous studies of patients without hypercalcemia [23–26]. In the RCA group, the estimated median filter lifespan was >72 h, which was comparable with the filter lifespan in previous studies [18–20]. In our present study, the average citrate dose was 2.52 mmol/L blood flow, which was lower than that in previous reports (3-4.3 mmol/L blood flow). The average postfilter ionCa level was 0.52 ± 0.22 mmol/L after 2 h of CVVH, which was higher than the recommended target (0.3-0.5 mmol/L) of RCA for CVVH [31]. Theoretically, the higher the citrate dose and the lower the postfilter ionCa are, the better the anticoagulation effect. According to a previous report, blood coagulation could be completely inhibited by ionCa between 0.25 mmol/L and 0.35 mmol/L. The anticoagulation effect of citrate was dose dependent, with ionCa between 0.33 mmol/L and 0.56 mmol/L [34]. The average postfilter ionCa of our present cohort was on the high end of the effect range, which was most likely one of the explanations of the long lifespan in our cohort. Additionally, all of the included patients had severe hypercalcemia with an average systemic ionCa of 1.37 ± 0.35 mmol/L after 2 h of CVVH. The average change between the systemic ionCa and postfilter ionCa was 0.85 ± 0.32 mmol/L in our RCA-CVVH patients, which was similar to the change between the systemic ionCa and postfilter ionCa in RCA-CVVH patients without hypercalcemia. This could potentially be another explanation for the long filter lifespan in our present cohort. In our opinion, the changes in ionCa before and after citrate anticoagulation might be highly important for the efficacy of citrate anticoagulation. Further studies are needed to verify this theory.

To the best of our knowledge, there have been no reports about the filter lifespan of LMWH anticoagulation in hypercalcemia patients. The efficacy of LMWH is not calcium dependent. Theoretically, there was no difference in the efficacy of LMWH anticoagulation between patients with and without hypercalcemia. The average LMWH dose of the LMWH-CVVH group in our present cohort was 38.8 IU/kg, which was consistent with the KDIGO guidelines and previous reports (33.5 IU/kg) of patients without hypercalcemia. The estimated median survival time of patients who received LMWH-CVVH was 24.0 h (IQR, 15.0–26.0), which was comparable with the results of previous reports of patients without hypercalcemia (26 h, IQR, 15–43) [35,36]. As reported in previous RCTs on patients without hypercalcemia, the filter lifespan of the LMWH-CVVH group was significantly shorter than that of the RCA-CVVH group in patients with severe hypercalcemia.

Furthermore, no obvious RCA-related complications, including citrate accumulation, metabolic acidosis, and metabolic alkalosis, were observed in the RCA-CVVH group of our present study. In patients with RCA-CVVH, citrate entered the systemic circulation and was mainly metabolized in the liver, kidney, and muscle in an oxygen-dependent manner [37]. None of the included patients in the RCA-CVVH group had impaired citrate metabolization. Therefore, it is understood that the risk of RCA-related complications is very low. The major complication of LMWH anticoagulation was the elevated bleeding risk. In our present study, the patients in the LMWH group tended to have more episodes of bleeding and blood transfusion than those in the RCA group, which was consistent with previous studies [23–26]. However, the difference between the two groups was not statistically significant, which was most likely caused by the relatively small sample size.

The reported in-hospital mortality rate of severe hypercalcemia patients who underwent intermittent hemodialysis was 25% [8],which was higher than the average in-hospital mortality rate of our present cohort (15.2%). Additionally, there was a slight reduction in the in-hospital mortality rate in the RCA group. However, the difference in in-hospital mortality between the RCA and LMWH groups was not significant. The mortality benefit of different hemodialysis modalities and different anticoagulation strategies needs further evaluation.

Our present study has several limitations. First, the retrospective nature should be considered one of the limitations. Although all of the important parameters were recorded during = CVVH treatment and the results were adjusted in multivariate analysis, the conclusions could potentially be biased by unobserved variables. Second, the sample size of our present study was relatively small. As mentioned above, the prevalence of severe hypercalcemia was very low. To the best of our knowledge, our present cohort was the largest cohort of severe hypercalcemia patients who underwent CVVH. Further multicenter studies with prospective designs are warranted to validate our findings.

## Conclusions

Our present study showed that, for severe hypercalcemia patients, RCA CVVH more effectively lowered calcium and led to a longer filter lifespan than LMWH-anticoagulation CVVH. For severe hypercalcemia patients who underwent CVVH treatment, citrate anticoagulation might be better than LMWH anticoagulation. Further multicenter studies with larger sample sizes, prospective designs, and randomized assignments are warranted to obtain stronger evidence.

## Data Availability

The datasets used and/or analyzed during the current study are available from the corresponding author on reasonable request.

## Abbreviations

AKI: acute kidney injury
APACHE II: Acute Physiology and Chronic Health Evaluation II
CRRT: continuous renal replacement therapy
CVVH: continuous venovenous hemofiltration
LMWH: low molecular weight heparin
RCA: regional citrate anticoagulation
SOFA: Sequential Organ Failure Assessment
